# Supplementary material for: CTF-based soft touch actuator for playing electronic piano
Source: Nat Commun. 2020 Oct 23;11:5358. doi: 10.1038/s41467-020-19180-3 (PMC7585428; doi:10.1038/s41467-020-19180-3)
Supplement: Supplementary file 2 — Supplementary Information [file 41467_2020_19180_MOESM2_ESM.pdf]

Supplementary Information

Title

CTF-based Soft Touch Actuator for Playing Electronic Piano

Authors

Manmatha Mahato,[§] Rassoul Tabassian,[§] Van Hiep Nguyen, Saewoong Oh, Sanghee Nam, Won-Jun Hwang, and Il-Kwon Oh[*]

Affiliations

National Creative Research Initiative for Functionally Antagonistic Nano-Engineering, Department of Mechanical Engineering, Korea Advanced Institute of Science and Technology (KAIST), 291 Daehak-ro, Yuseong-gu, Daejeon 34141, Republic of Korea.

[§] These authors contributed equally to this work.

[*] Corresponding author. Email: ikoh@kaist.ac.kr

Contents

Supplementary Figure 1. Structural characterization of PIM-1. **(a)** ^{1}H NMR spectra on $CDCl_3$, and **(b)** N_2 adsorption-desorption isotherms at 77 K.

Supplementary Figure 2. Thermogravimetric and N_2-sorption isotherm analysis. (a) TG-DTG spectra of PIM-1, TP4, TP5, and TP6. Inset: TG spectra of TP7. **(b)** Nitrogen adsorption-desorption isotherms of TP6 and TP7 at 77 K. Filled and empty symbols denote adsorption and desorption, respectively. Inset: respective specific surface area data. All the measurements were carried out twice using 200 mg of each CTFs as mentioned.

Supplementary Figure 3. Morphological characterization from lower to higher magnification. **(a-c)** High resolution SEM images of TP4. **(d-f)** High resolution SEM images of TP5.

Supplementary Figure 4. Morphological and Ar-sorption isotherm analysis of TP6. (a) Magnified SEM image. **(b)** Argon adsorption-desorption isotherm at 87 K, displays microporous nature of it with hysteretic *Type-1* sorption isotherm.

Supplementary Figure 5. FT-IR spectra (wavenumber: 3100 to 2600 cm^{-1}) of PIM-1 and corresponding novel PIM-1 based CTFs to show the presence of C-H moiety.

Supplementary Figure 6. Solid-state ^{1}H CP-MAS NMR spectroscopic analysis of PIM-1, TP4, TP5, and TP6 CTFs.

Supplementary Figure 7. Solid-state CP-MAS NMR spectra of CTFs. (a) ^{13}C CP-MAS NMR spectra of TP4, TP5, and TP6 CTFs. **(b)** Interpretation of ^{13}C CP-MAS NMR signals of TP4 CTFs.

Supplementary Figure 8. XPS spectra of TP4, TP5, and TP6 CTFs. Deconvoluted XPS spectra of C1s configuration of **(a)** TP4, **(b)** TP5, and **(c)** TP6 CTFs.

Supplementary Figure 9. Elemental composition analyses of CTFs. **(a)** High resolution tomography (STEM-HAADF) image of TP6 with the corresponding **(b)** carbon, **(c)** nitrogen, **(d)** oxygen, and **(e)** overlapped carbon-nitrogen-oxygen elemental mapping images. **(f)** XPS survey spectra of TP4, TP5, and TP6 CTFs.

Supplementary Figure 10. Possible mechanism for high charge storage capacity of novel CTFs and synthesis of PIM-1 and corresponding CTFs. (a) Accommodation of positive (A-B) and negative (A-C) ions in presence of dibenzo-*p*-dioxine unit. **(b)** Synthetic route of PIM-1 with proposed chemical structure. **(c)** Synthetic route of CTFs along with its proposed chemical structure.

Supplementary Figure 11. Electrochemical impedance spectra (EIS) of TP4PP, TP5PP, and TP6PP ionic soft actuators. Inset: The magnified high frequency region.

Supplementary Figure 12. Electrochemical and actuation performances of air-working ionic soft actuators. **(a)** Stability of CV responses up to 20 cycles for TP6PP soft actuator. **(b)** Linear relationship of bending displacements with applied potentials (0.1-1.0 V) for TP6PP soft actuator. **(c)** Durability of long-term actuation performances of TP6PP. **(d)** Time-dependent bending responses of PP, TP4PP, TP5PP, and TP6PP soft actuators under ± 1.0 V square sine wave input voltages at frequency of 0.1 Hz. **(e)** Time-dependent bending responses of TP4PP ionic soft actuator under ± 1.0 V sine wave input voltages at frequency of 0.1 Hz. **(f)** Time-dependent bending responses of TP5PP ionic soft actuator under ± 1.0 V sine wave input voltages at frequency of 0.1 Hz.

Supplementary Figure 13. Circuit diagram of robotic hand for controlling air-working ionic soft actuators.

Supplementary Table 1. Elemental (CHNO) analysis of PIM-1-based CTFs (TP4, TP5, and TP6).

Supplementary Table 2. Repeatability of electrochemical CV analysis for TP4, TP5, and TP6 CTFs in aqueous and non-aqueous electrolytes as different scan rates up to three consecutive cycles.

Supplementary Table 3. Mechanical properties of actuator layers.

Supplementary Table 4. Comparison of bending performance of ionic soft actuators.

Supplementary Table 5. Blocking forces of PP and TP6PP ionic soft actuators.

Supplementary Movie 1. Robotic fingers array taking lead in playing piano app.

Supplementary Movie 2. Robotic fingers playing piano for “Happy Birthday” song.

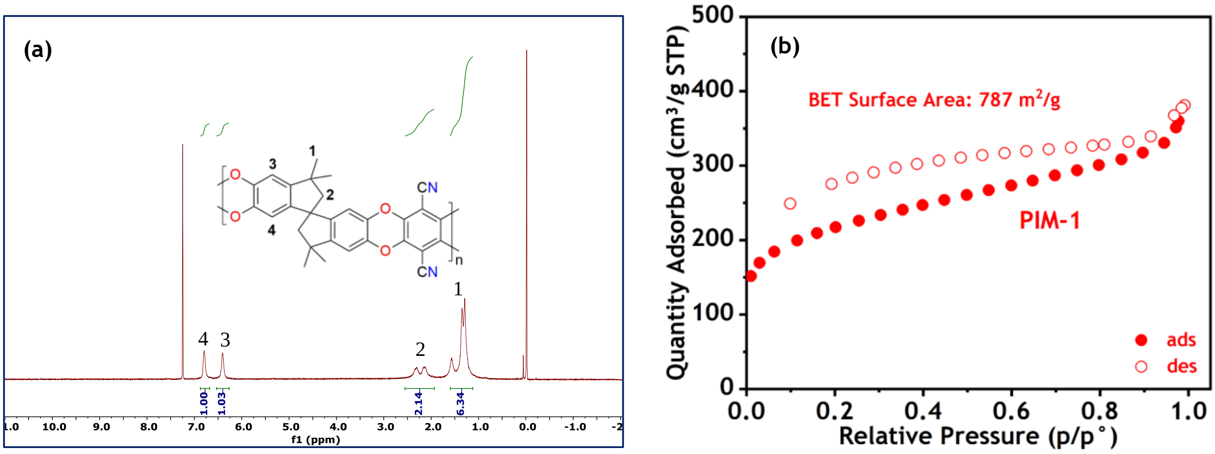

Supplementary Figure 1. Structural characterization of PIM-1. **(a)** ^1H NMR spectra on $CDCl_3$, and **(b)** N_2 adsorption-desorption isotherms at 77 K.

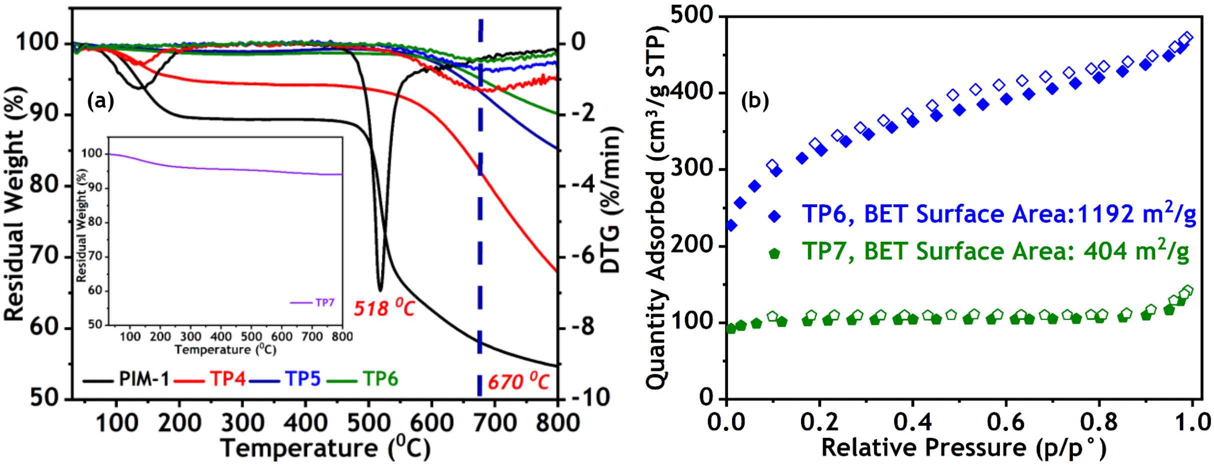

Supplementary Figure 2. Thermogravimetric and N_2-sorption isotherm analysis. (a) TG-DTG spectra of PIM-1, TP4, TP5, and TP6. Inset: TG spectra of TP7. **(b)** Nitrogen adsorption-desorption isotherms of TP6 and TP7 at 77 K. Filled and empty symbols denote adsorption and desorption, respectively. Inset: respective specific surface area data. All the measurements were carried out twice using 200 mg of each CTFs as mentioned.

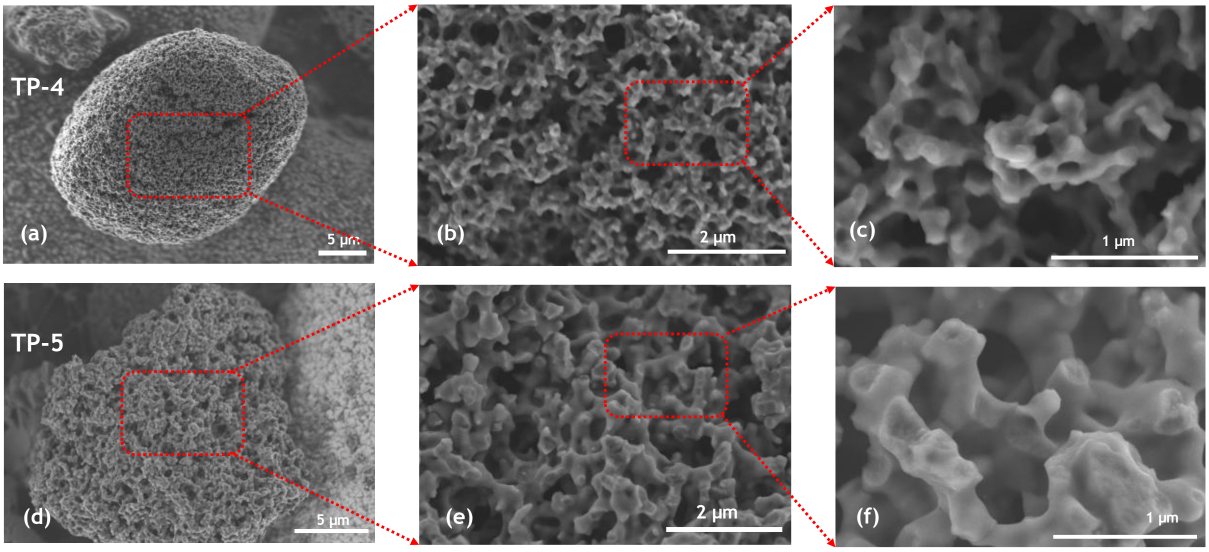

Supplementary Figure 3. Morphological characterization from lower to higher magnification. **(a-c)** High resolution SEM images of TP4. **(d-f)** High resolution SEM images of TP5.

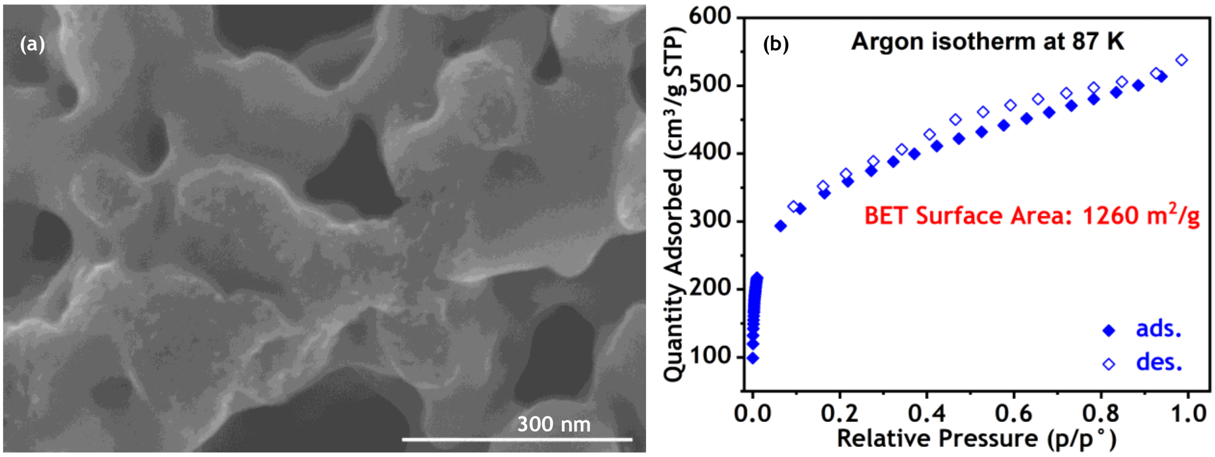

Supplementary Figure 4. Morphological and Ar-sorption isotherm analysis of TP6. (a) Magnified SEM image. **(b)** Argon adsorption-desorption isotherm at 87 K, displays microporous nature of it with hysteretic *Type-1* sorption isotherm.

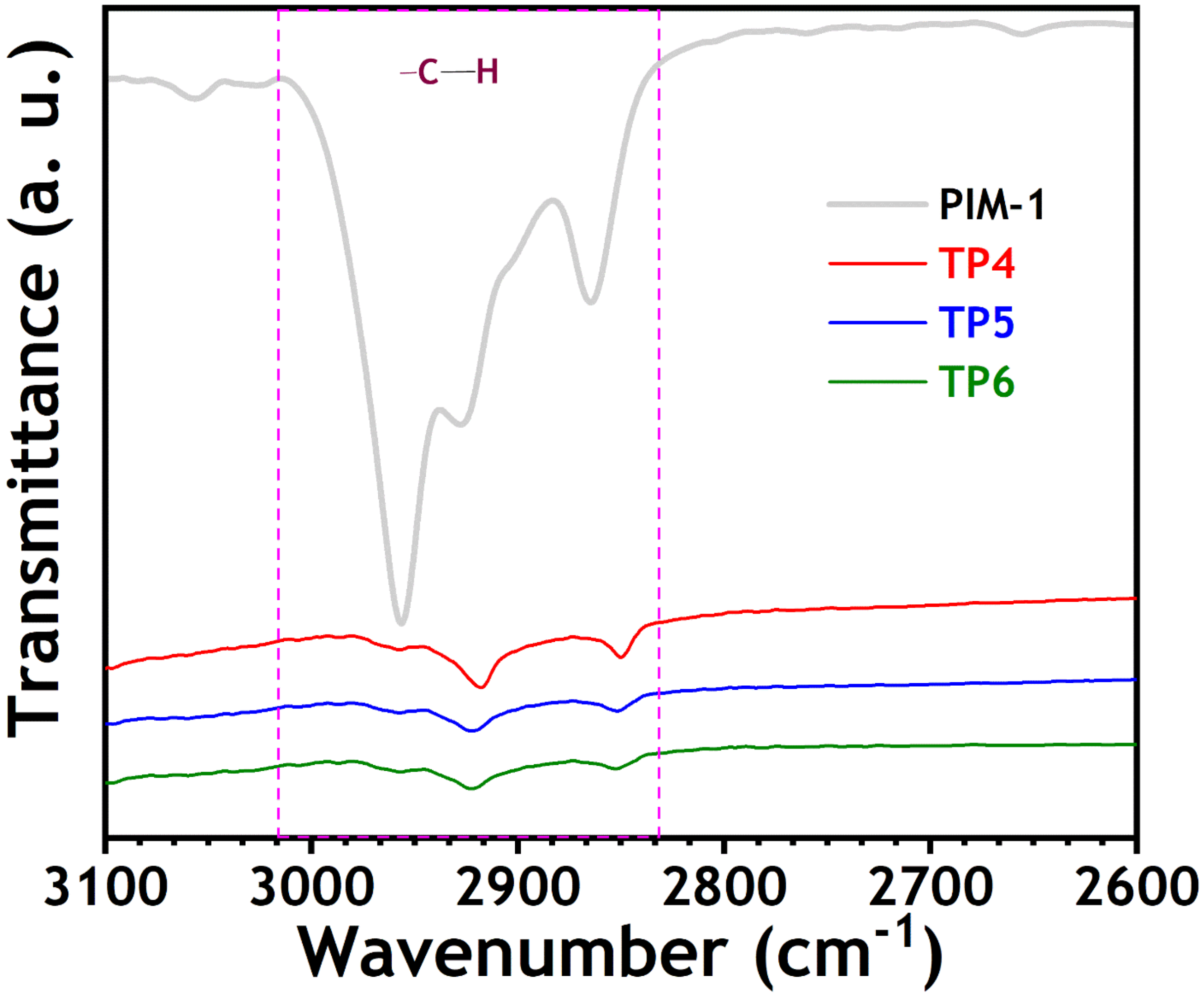

Supplementary Figure 5. FT-IR spectra (wavenumber: 3100 to 2600 cm^{-1}) of PIM-1 and corresponding novel PIM-1 based CTFs to show the presence of C-H moiety.

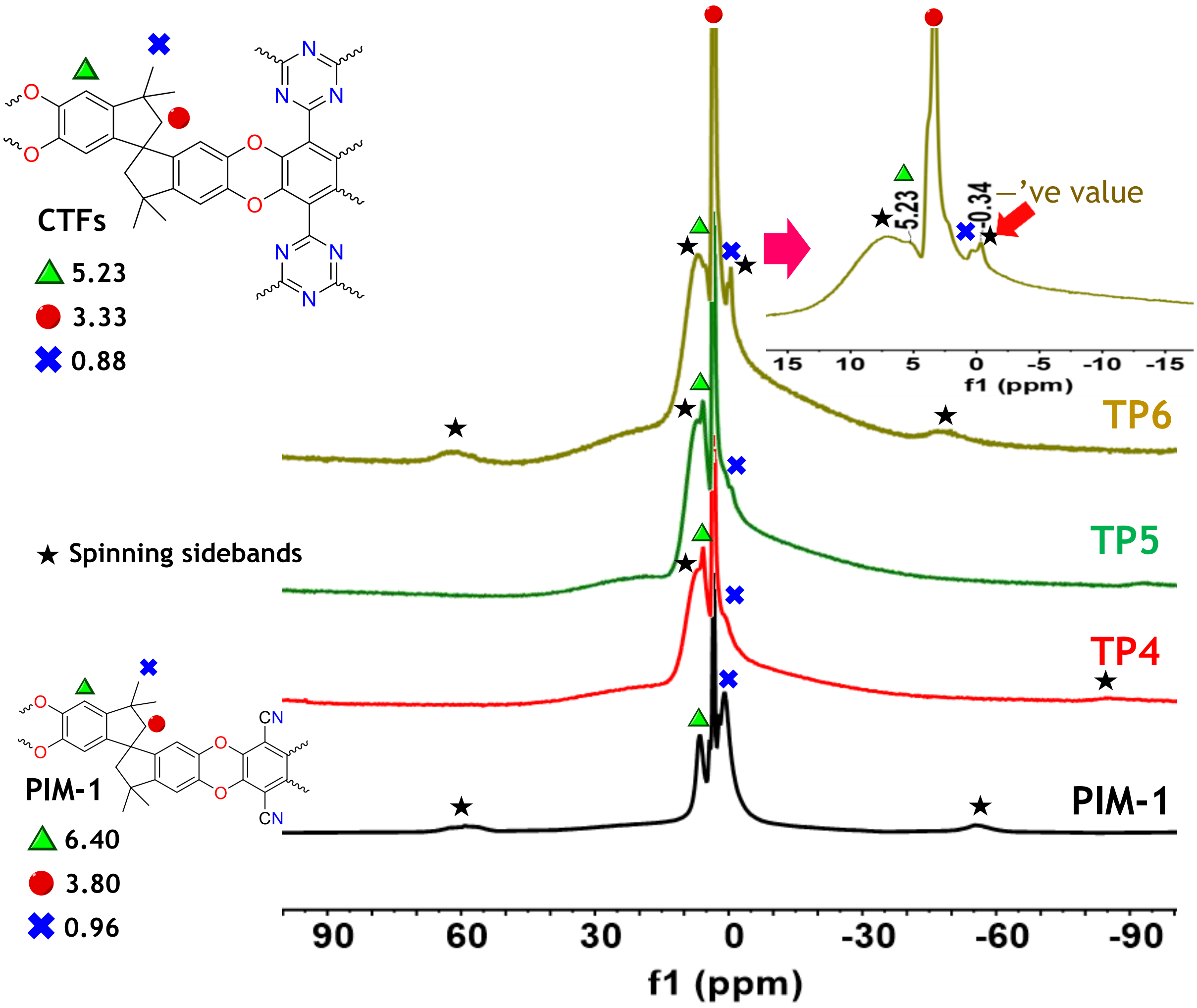

Supplementary Figure 6. Solid-state ^{1}H CP-MAS NMR spectroscopic analysis of PIM-1, TP4, TP5, and TP6 CTFs.

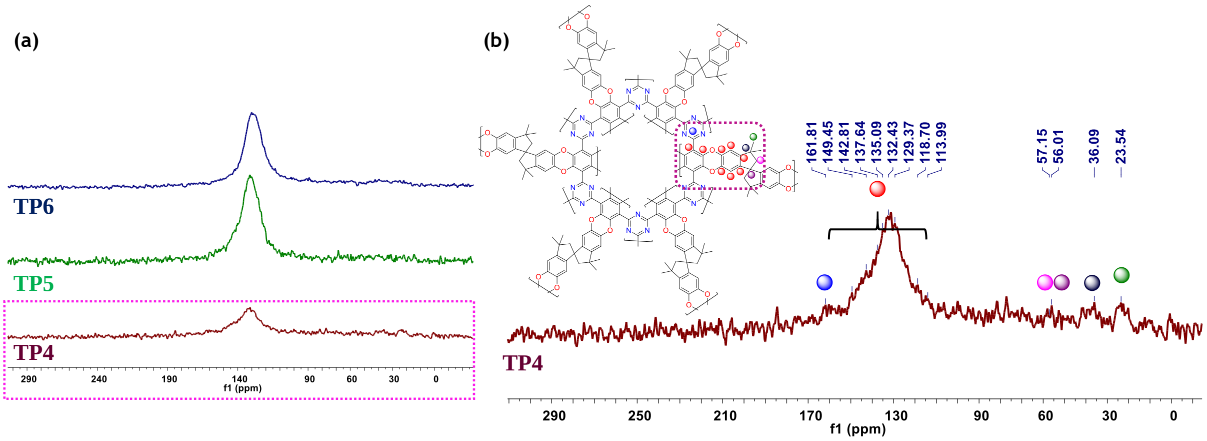

Supplementary Figure 7. Solid-state CP-MAS NMR spectra of CTFs. (a) ^{13}C CP-MAS NMR spectra of TP4, TP5, and TP6 CTFs. **(b)** Interpretation of ^{13}C CP-MAS NMR signals of TP4 CTFs.

The integrity of framework structures of TP4, TP5, and TP6 CTFs is also reflected in the solid-state ^{13}C CP-MAS NMR spectrum (Supplementary Fig. 7a, b). There are mainly six different types of chemically non-equivalent carbons present in the reported CTFs which are highlighted by spherical symbols with individual colours (Supplementary Fig. S7b: Inset). The obtained NMR signal at 161 ppm confirms the presence of triazine ring carbon in the reported CTFs,[1] while the signals in between 129-149 ppm are corresponding to the phenyl ring carbons of as present modified PIM-1 structures. The NMR signal at 118 ppm is related to the –C-O (-C–**C**-O-) which is connected to the aromatic benzene ring. The signal at 113 ppm is characteristic NMR signal for nitrile groups as well as the carbon of the phenyl ring to which the nitrile groups are connected. The presence of four different types of alkyl carbons with/without protons appeared as broad NMR signals in between 23-57 ppm is mainly due to the high temperature synthetic procedures. The obtained NMR signals of those alkyl carbons also support the retaining of frameworks on the reported CTFs structures. Additionally, it is also observed from Supplementary Fig. S7a that with the increase of synthetic temperature from 400-600 ˚C, the characteristic NMR signals of specified carbons became weak which support the increase extent of carbonization/graphitization at elevated temperature.

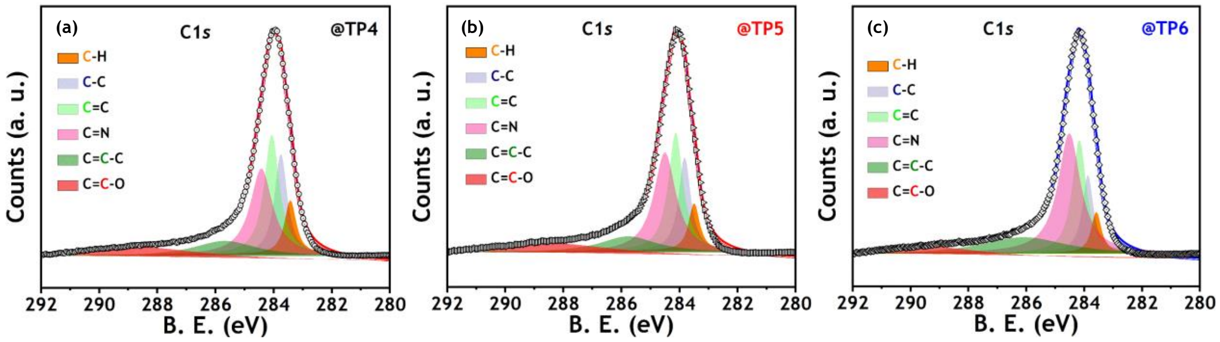

Supplementary Figure 8. XPS spectra of TP4, TP5, and TP6 CTFs. Deconvoluted XPS spectra of C1*s* configuration of **(a)** TP4, **(b)** TP5, and **(c)** TP6 CTFs.

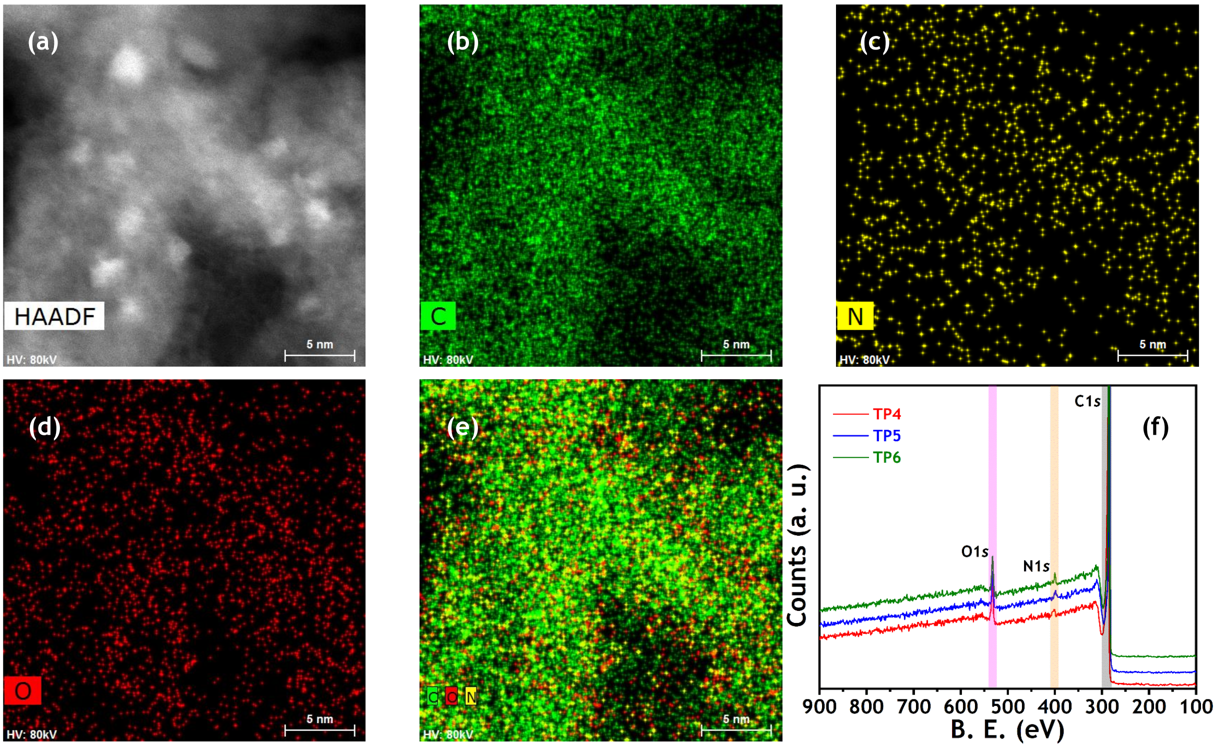

Supplementary Figure 9. Elemental composition analyses of CTFs. **(a)** High resolution tomography (STEM-HAADF) image of TP6 with the corresponding **(b)** carbon, **(c)** nitrogen, **(d)** oxygen, and **(e)** overlapped carbon-nitrogen-oxygen elemental mapping images. **(f)** XPS survey spectra of TP4, TP5, and TP6 CTFs.

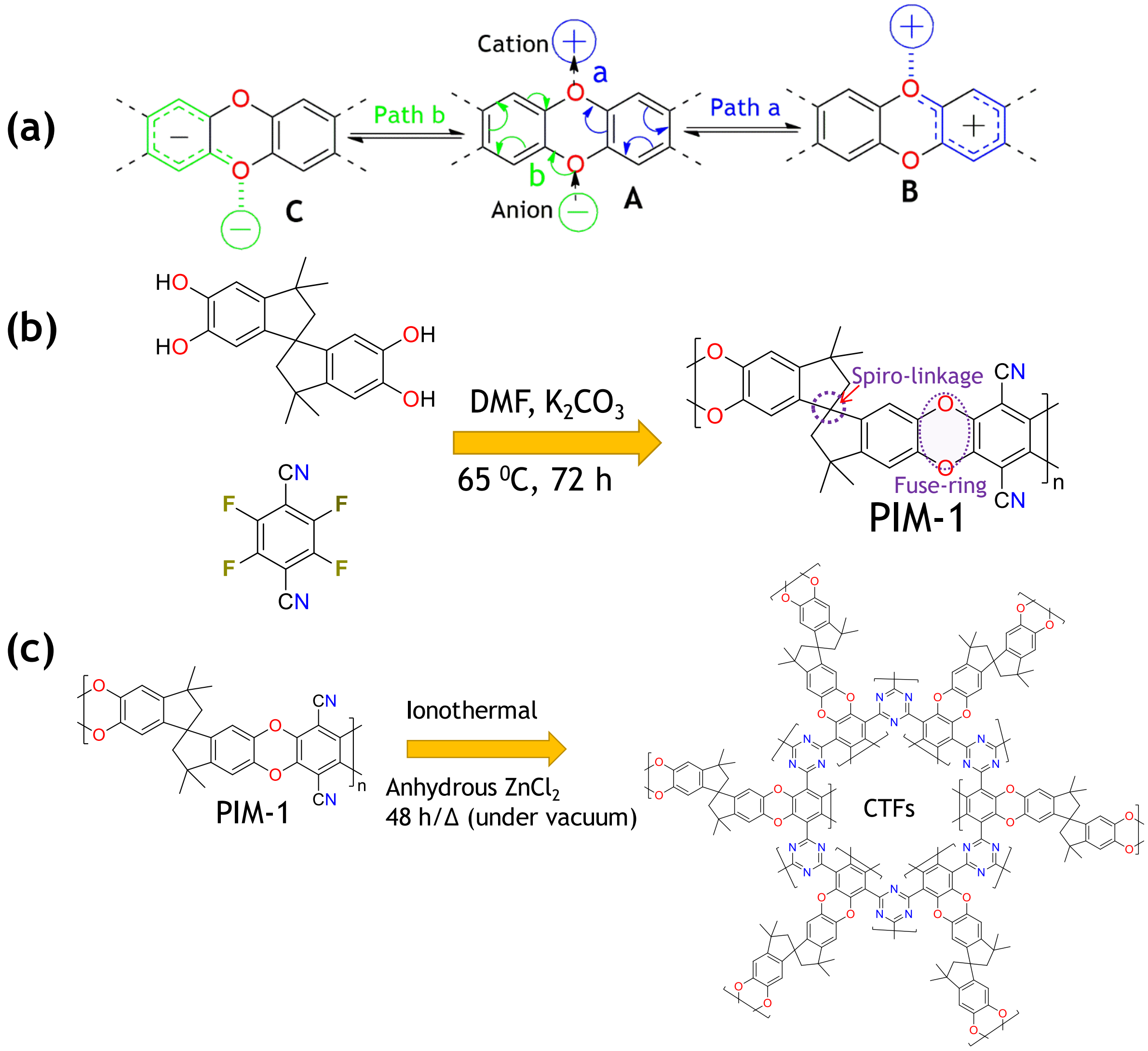

Supplementary Figure 10. Possible mechanism for high charge storage capacity of novel CTFs and synthesis of PIM-1 and corresponding CTFs. (a) Accommodation of positive (A-B) and negative (A-C) ions in presence of dibenzo-*p*-dioxine unit. **(b)** Synthetic route of PIM-1 with proposed chemical structure. **(c)** Synthetic route of CTFs along with its proposed chemical structure.

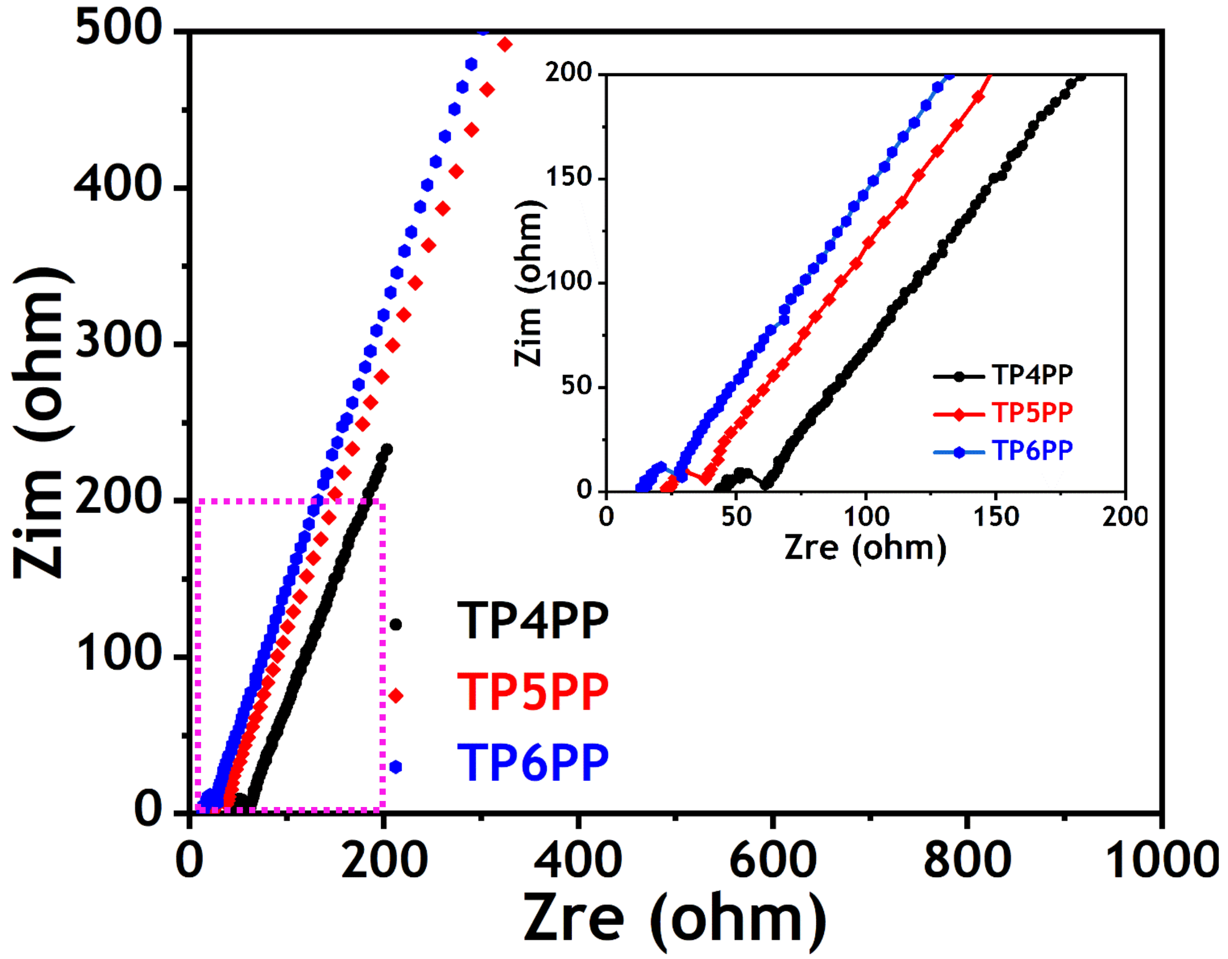

Supplementary Figure 11. Electrochemical impedance spectra (EIS) of TP4PP, TP5PP, and TP6PP ionic soft actuators. Inset: The magnified high frequency region.

A Nyquist plot of all CTFs-based actuators shows semi-circles at high frequency region related to charge transfer resistance (R_{ct}) and a straight-line at low frequency region related to solid-state diffusion of ions. It has been observed from the spectra that TP6PP actuator shows smallest R_{ct} value (28 ohms) than those of the TP5PP (39 ohms) and TP4PP (63 ohms) actuators. These results also support the higher order of charge transport in the TP6PP actuator in comparison to others and correspondingly TP6PP shows high areal capacitance.

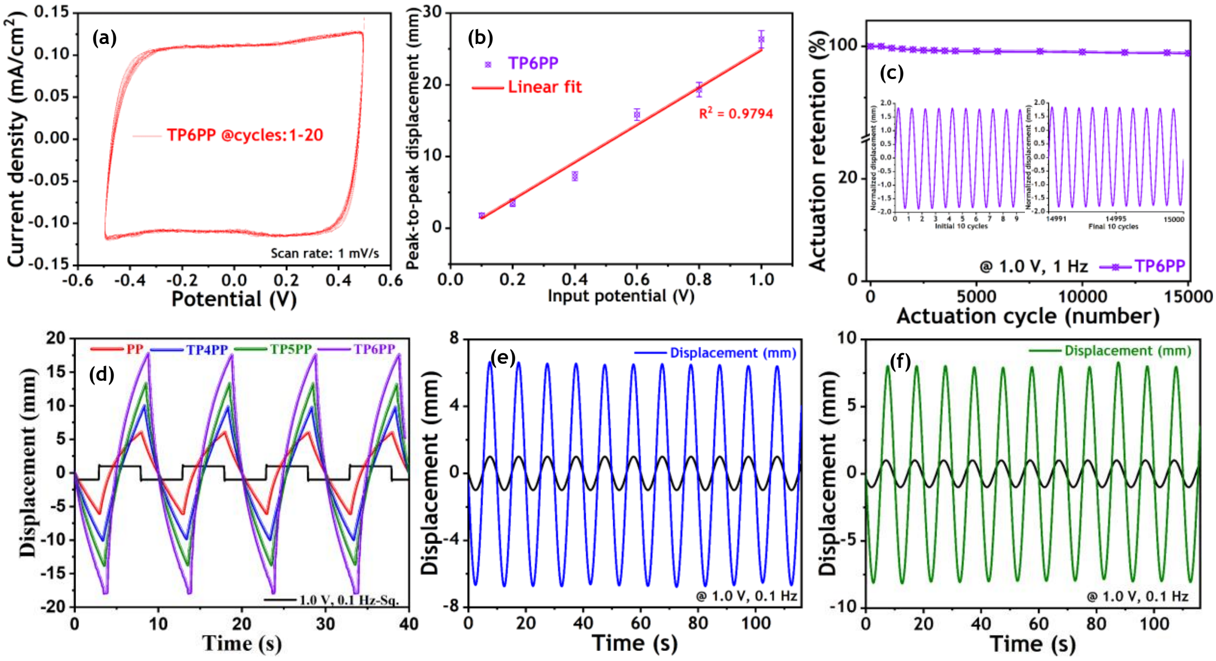

Supplementary Figure 12. Electrochemical and actuation performances of air-working ionic soft actuators. **(a)** Stability of CV responses up to 20 cycles for TP6PP soft actuator. **(b)** Linear relationship of bending displacements with applied potentials (0.1-1.0 V) for TP6PP soft actuator. **(c)** Durability of long-term actuation performances of TP6PP. **(d)** Time-dependent bending responses of PP, TP4PP, TP5PP, and TP6PP soft actuators under ± 1.0 V square sine wave input voltages at frequency of 0.1 Hz. **(e)** Time-dependent bending responses of TP4PP ionic soft actuator under ± 1.0 V sine wave input voltages at frequency of 0.1 Hz. **(f)** Time-dependent bending responses of TP5PP ionic soft actuator under ± 1.0 V sine wave input voltages at frequency of 0.1 Hz.

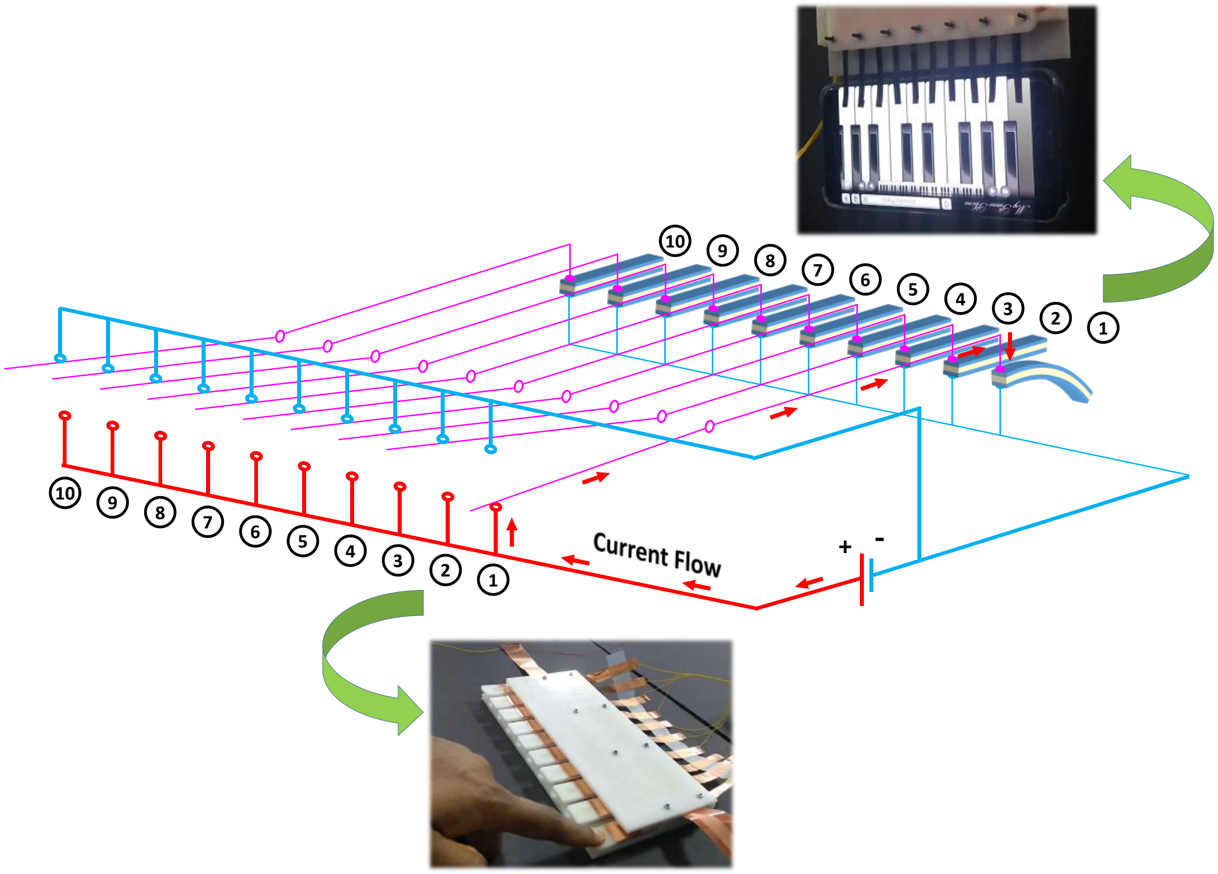

Supplementary Figure 13. Circuit diagram of robotic hand for controlling air-working ionic soft actuators.

Supplementary Table 1. Elemental (CHNO) analysis of synthesized PIM-1 based CTFs (TP4, TP5, and TP6).

CHN-O Analyses		% C	% O	% H	% N
Theoretically		74.06	13.15	4.14	8.64
Experimentally	**TP4**	80.40	7.60	2.70	9.30
	**TP5**	80.80	6.20	1.60	11.40
	**TP6**	81.90	5.90	1.80	10.40

Theoretical calculations were done by considering one repeating unit of CTFs.

Formula: $C_{180}H_{120}N_{18}O_{24}$
Formula weight: 2918.88 (100%)
Elemental Analysis (**Wt.%**): **C**, 74.06; **H**, 4.14; **N**, 8.64; **O**, 13.15

Supplementary Table 2. Repeatability of electrochemical CV analysis for TP4, TP5, and TP6 CTFs in aqueous and non-aqueous electrolytes as different scan rates up to three consecutive cycles.

Electrolyte	CTFs	Specific capacitance (F/g)											
		Scan rate (V/s)											
		0.01				0.05				0.1			
		Test-1	Test-2	Test-3	**Ave.**	Test-1	Test-2	Test-3	**Ave.**	Test-1	Test-2	Test-3	**Ave.**
H_2SO_4, 1(M)	TP6	525	529	513	**522**	476	465	468	**470**	452	457	449	**453**
	TP5	306	296	303	**301**	257	268	261	**262**	231	234	245	**237**
	TP4	221	228	219	**223**	210	207	203	**206**	187	194	185	**189**
KOH, 1(M)	TP6	340	331	339	**337**	317	313	321	**317**	292	307	294	**298**
	TP5	245	238	251	**244**	225	213	215	**218**	207	201	197	**202**
	TP4	148	137	145	**143**	120	134	126	**127**	109	122	117	**116**
EMIM-BF_4 (0.5M)	TP6	463	471	468	**467**	441	438	449	**443**	411	417	419	**416**
	TP5	317	307	313	**312**	295	284	289	**289**	258	274	263	**265**
	TP4	162	166	175	**167**	135	147	138	**140**	133	124	132	**129**

Repeatability of electrochemical performances at three different scan rates (0.01V/s, 0.05V/s, and 0.1V/s) is performed and the obtained specific capacitance data are shown in Supplementary Table 2. It is observed that high scan rate reduces the capacitance value which is quite common in microporous organic polymer electrode materials.[2]

Supplementary Table 3. Mechanical properties of actuator layers.

Membrane	Young's modulus, MPa	Tensile strength, MPa	Elongation at break, %
Nafion	24.10	1.36	10.89
PEDOT-PSS	36.01	3.62	35.20
PEDOT-PSS-CTF (TP6)	48.56	4.85	31.52

It has been observed that PEDOT-PSS-CTF (TP6) exhibited highest tensile strength and Young's modulus than the other layers used for the fabrication of CTFs-based ionic soft actuators. This implies the strong intermolecular interaction in between PEDOT-PSS and CTFs. This observed data also supports the proposed mechanism (Fig. 4g-C, main manuscript) for the electronic interaction of CTFs with PEDOT-PSS towards the increase of surface charge and electric conductivity.

Supplementary Table 4. Comparison of bending performance of ionic soft actuators.

Ionic soft actuator	Input potential (sine wave) & frequency	Length, mm	Thickness, µm	Bending displacement (peak-to-peak), mm	Ref.
3D G–CNT–Ni/PP	±1.0 V and 0.1 Hz	24	248	4.84	3
PP/3D GCN–NG	±0.5 V and 0.1 Hz	18	100	6.50	4
GM-NG	±3.0 V and 0.1 Hz	-	-	6.40	5
BS-COF-C900/PP	±0.5 V and 0.1 Hz	20	85	8.60	6
Th-SNG/PP	±0.5 V and 0.1 Hz	26	90	4.60	7
HPNC-900/PP	±0.5 V and 0.1 Hz	20	100	6.99	8
*p*MoS2-*n*SN*r*GO nanohybrid	±0.5 V and 0.1 Hz	20	105	9.90	9
HLrGOP	±5.0 V and 0.1 Hz	20	-	8.52	10
PS-*b*-PSS-EMIm/EMImBF4 electrolyte based NS co-doped graphene/PP electrodes	±0.5 V and 0.1 Hz	20	80	6.80	11
IL-IPMC	±10.0 V and 0.1 Hz	30	313	11.10	12
Nacre-based carbon nanomeshes electrode	±3.0 V (Sq.) and 0.1 Hz	30	165	10.00	13
BP-CNTs/CNTs	±1.5 V (Sq.), 0.1 Hz	23	115	12.50	14
Nafion and Pt based IPMC	±1.0 V, 0.1 Hz	25	-	2.00	15
**TP6PP**	**±0.5 V and 0.1 Hz**	**25**	**115**	**13.50**	**This work**

The width of the fabricated ionic soft actuators used in this research article is 3.0 ± 0.017 mm.

PP: PEDOT-PSS

Supplementary Table 5. Blocking forces of PP and TP6PP ionic soft actuators.

Voltage (V)	PP Actuator			TP6PP Actuator			Improvement %
	Average blocking force (mN)	Standard error (mN)	Normalized blocking force	Average blocking force (mN)	Standard error (mN)	Normalized blocking force	
0.2	0.309	0.012	2.425	0.620	0.004	5.445	124.5
0.4	0.620	0.006	4.851	1.072	0.007	9.423	94.2
0.6	0.882	0.004	6.903	1.454	0.011	12.774	85.0
0.8	1.048	0.007	8.209	1.716	0.002	15.078	83.7
1.0	1.215	0.009	9.515	2.050	0.006	18.010	89.3
1.2	1.287	0.002	10.075	2.240	0.007	19.685	95.4
1.4	1.358	0.002	10.635	2.550	0.003	22.407	110.7
1.6	1.406	0.010	11.008	2.621	0.001	23.035	109.3
1.8	1.454	0.006	11.381	2.693	0.004	23.663	107.9
2.0	1.477	0.011	11.567	2.788	0.008	24.501	111.8
						Average	101.2

Normalized blocking forces were calculated by dividing the average blocking forces to the weight of the actuators. Each measurement was performed four times. The improvement percentage is calculated by the ratio between PP and TP6PP actuators.

- Mass of PP actuator = 13.02 mg (Weight = 0.128 mN)
- Mass of TP6PP actuator = 11.60 mg (Weight = 0.114 mN)

References

1. Dey, S., Bhunia, A., Esquivel, D. and Janiak, C. Covalent triazine-based frameworks (CTFs) from triptycene and fluorene motifs for CO_2 adsorption. J. Mater. Chem. A, **4**, 6259-6263 (2016).

2. Kou, Y., Xu, Y., Guo, Z. and Jiang, D. Supercapacitive Energy Storage and Electric Power Supply Using an Aza-Fused π-Conjugated Microporous Framework. Angew. Chem. In. Ed. 123, 8912-8916 (2011).

3. Kim, J., Bae, S.H., Kotal, M., Stalbaum, T., Kim, K. J. and Oh, I. K. Soft but Powerful Artificial Muscles Based on 3D Graphene-CNT-Ni Heteronanostructures. Small **13**, 1701314, (2017).

4. Kotal, M., Kim, J., Tabassian, R., Roy, S., Nguyen, V. H., Koratkar, N. and Oh, I. K. Highly Bendable Ionic Soft Actuator Based on Nitrogen-Enriched 3D Hetero-Nanostructure Electrode. Adv. Funct. Mater. **28**, 1802464 (2018).

5. Tabassian, R., Kim, J., Nguyen, V. H., Kotal, M. and Oh, I. K. Functionally Antagonistic Hybrid Electrode with Hollow Tubular Graphene Mesh and Nitrogen-Doped Crumpled Graphene for High-Performance Ionic Soft Actuators. Adv. Funct. Mater. **28**, 1705714 (2018).

6. Roy, S., Kim, J., Kotal, M., Tabassian, R., Kim, K. J. and Oh, I. K. Collectively Exhaustive Electrodes Based on Covalent Organic Framework and Antagonistic Co-Doping for Electroactive Ionic Artificial Muscles. Adv. Funct. Mater. **29,** 1900161 (2019).

7. Kotal, M., Kim, J., Kim, K. J. and Oh, I. K. Sulfur and Nitrogen Co-Doped Graphene Electrodes for High-Performance Ionic Artificial Muscles. Adv. Mater. **28**, 1610-1615 (2016).

8. Roy, S., Kim, J., Kotal, M., Kim, K. J. and Oh, I. K. Electroionic Antagonistic Muscles Based on Nitrogen-Doped Carbons Derived from Poly (Triazine-Triptycene). Adv. Sci. **4**, 1700410 (2017).

9. Manzoor, M. T., Nguyen, V. H., Umrao, S., Kim, J. H., Tabassian, R., Kim, J. E. and Oh, I. K. Mutually Exclusive p-Type and n-Type Hybrid Electrode of MoS_2 and Graphene for Artificial Soft Touch Fingers. Adv. Funct. Mater. **29**, 1905454 (2019).

10. Kim, J., Jeon, J. H., Kim, H. J., Lim, H. and Oh, I. K. Durable and water-floatable ionic polymer actuator with hydrophobic and asymmetrically laser-scribed reduced graphene oxide paper electrodes. ACS Nano **8**, 2986-2997 (2014).

11. Nguyen, V. H., Kim, J., Tabassian, R., Kotal, M., Jun, K., Oh, J. H., Son, J. M., Manzoor, M. T., Kim, K. J. and Oh, I. K. Electroactive Artificial Muscles Based on Functionally Antagonistic Core–Shell Polymer Electrolyte Derived from PS-b-PSS Block Copolymer. Adv. Sci. **6**, 1801196 (2019).

12. Guo, D., Han, Y., Huang, J., Meng, E., Ma, L., Zhang, H. and Ding, Y. Hydrophilic poly (vinylidene fluoride) film with enhanced inner channels for both water-and ionic liquid-driven ion-exchange polymer metal composite actuators. ACS Appl. Mater. & Inter. **11**, 2386-2397, (2019).

13. Han, X., Kong, M., Li, M., Li, X., Yang, W. and Li, C. Nacre-based carbon nanomeshes for soft ionic actuator with large and rapid deformation. J. Mater. Chem. C, DOI: 10.1039/c9tc06186j, (2020).

14. Wu, G., Wu, X., Xu, Y., Cheng, H., Meng, J., Yu, Q., Shi, X., Zhang, K., Chen, W. and Chen, S. High-Performance Hierarchical Black-Phosphorous-Based Soft Electrochemical Actuators in Bioinspired Applications. Adv. Mater. 1806492 (2019).

[15] Ma, S., Zhang, Y., Liang, Y., Ren, L., Tian, W. and Ren, L. High-Performance Ionic-Polymer–Metal Composite: Toward Large-Deformation Fast-Response Artificial Muscles. Adv. Funct. Mater. 1908508, (2019).
