# Supplementary material for: CTF-based soft touch actuator for playing electronic piano
Source: Nat Commun. 2020 Oct 23;11:5358. doi: 10.1038/s41467-020-19180-3 (PMC7585428; doi:10.1038/s41467-020-19180-3)
Supplement: Supplementary file 4 — Description of Additional Supplementary Files [file 41467_2020_19180_MOESM4_ESM.pdf]

Description of Additional Supplementary Files

File Name: Supplementary Movie 1
Description: **Keyboard test for artificial robotic touch fingers.** Connection interface bewteen human fingers and robotic fingers was tested with artificial robotic touch array.

File Name: Supplementary Movie 2
Description: **Playing electronic piano by artificial finger array.** Soft touch finger array was used to play electronic piano for "happy birthday" song.
